# Trefftz co-chain calculus

**DOI:** 10.1007/s00033-021-01671-y

**Published:** 2022-01-25

**Authors:** Daniele Casati, Lorenzo Codecasa, Ralf Hiptmair, Federico Moro

**Affiliations:** 1 Seminar for Applied Mathematics, ETH Zurich, Zurich, Switzerland; 2 Dipartimento di Elettronica, Informazione e Bioingegneria, Politecnico di Milano, Milan, Italy; 3 Dipartimento di Ingegneria Industriale, Università degli Studi di Padova, Padua, Italy

**Keywords:** Co-chain calculus, Finite element exterior calculus, Discrete exterior calculus, Trefftz method, 65N30, 65N80, 35Q61

## Abstract

We are concerned with a special class of discretizations of general linear transmission problems stated in the calculus of differential forms and posed on ${\mathbb {R}}^n$. In the spirit of domain decomposition, we partition ${\mathbb {R}}^n=\Omega \cup \Gamma \cup \Omega _{+}$, $\Omega$ a bounded Lipschitz polyhedron, $\Gamma :=\partial \Omega$, and $\Omega _{+}$ unbounded. In $\Omega$, we employ a mesh-based discrete co-chain model for differential forms, which includes schemes like finite element exterior calculus and discrete exterior calculus. In $\Omega _{+}$, we rely on a meshless Trefftz–Galerkin approach, i.e., we use special solutions of the homogeneous PDE as trial and test functions. Our key contribution is a unified way to couple the different discretizations across $\Gamma$. Based on the theory of discrete Hodge operators, we derive the resulting linear system of equations. As a concrete application, we discuss an eddy-current problem in frequency domain, for which we also give numerical results.

## Boundary value problems

### Linear partial differential equations in exterior calculus

The calculus of differential forms, also known as *exterior calculus*, is a powerful tool for expressing a host of apparently different linear boundary value problems in a unifying way through *differential forms*; see [23, p. 265, Sections 1 and 2]. Concretely, we write $\Lambda ^{k}({\mathbb {R}}^{n})$ for the space of differential forms of order *k*, $0\le k\le n$, $n\in {\mathbb {N}}^*$, in ${\mathbb {R}}^{n}$ [8, p. 13, Section 2.2].

Each member of the family of partial differential equations we are concerned with in this work can naturally be split into two sets of equations. The first involves the exterior derivative operator $\mathrm {d}$ and comprises the *equilibrium equations*

1.1a $$\begin{aligned}&\left\{ \begin{array}{rcl} \mathrm {d}u &{}=&{} \left( -1\right) ^l \varvec{\sigma } {,}\\ \mathrm {d}{\mathbf {j}}&{}=&{} \psi - \psi _0 {,}\end{array}\right.&\quad \text {in }{\mathbb {R}}^n, \end{aligned}$$

connecting the differential forms $u\in \Lambda ^{l-1}({\mathbb {R}}^n)$, $\varvec{\sigma }\in \Lambda ^{l}({\mathbb {R}}^n)$, ${\mathbf {j}}\in \Lambda ^{m}({\mathbb {R}}^n)$, $\psi \in \Lambda ^{m+1}({\mathbb {R}}^n)$, and the known source $\psi _0\in \Lambda ^{m+1}({\mathbb {R}}^n)$ [25, p. 243, Table 2.1], given $l \in \{1,\ldots ,n\}$ and ${m :=n-l}$.

The other set is formed by the *constitutive equations*

1.1b $$\begin{aligned}&\left\{ \begin{array}{rcl} {\mathbf {j}}&{}=&{} \star _\alpha \varvec{\sigma } {,}\\ \psi &{}=&{} \star _\gamma u \,{,}\end{array}\right.&\quad \text {in }{\mathbb {R}}^n. \end{aligned}$$

The symbols $\star _\alpha$ and $\star _\gamma$ stand for *Hodge operators*, which supply linear mappings of *l*-forms into *m*-forms [8, p. 12]. These are induced by the *material metrics* $\alpha$ and $\gamma$, which are Riemannian metrics in the simplest case. However, $\alpha$ and $\gamma$ can be more general (pseudo-Riemannian) metrics. In particular, $\gamma$ can also be

- zero, which is the *static case*,
- negative, which is the case of a Helmholtz-type equation describing wave propagation, or
- imaginary, which leads to a dissipation problem, describing, e.g., eddy currents: this is the case of (1.2b).

If ${\mathbb {R}}^{n}$ is equipped with Cartesian coordinates, these metrics can be represented by invertible matrix fields.

#### Remark 1

Note that the equations are posed on the unbounded domain ${\mathbb {R}}^{n}$ and, thus, we have to impose suitable decay or radiation conditions for ${\mathbf {x}}\rightarrow \infty$ on the forms, or, equivalently, select the forms from weighted Sobolev spaces; see [39, p. 42, Section 1.4], [43, p. 1484, Section 4], [37, p. 602, Section 3]. The concrete conditions will depend on the signs of $\alpha$ and $\gamma$.

### Concrete case

Let us connect the general problem of Sect. 1.1 to a concrete boundary value problem relevant in computational electromagnetics, namely the *eddy-current problem* in frequency domain in three dimensions [1]. For (1.1), this amounts to the case $n=3$, $l=2$, and $m=1$. The electromagnetic fields involved—designated with the customary symbols—are the magnetic vector potential ${\mathbf {A}}:{\mathbb {R}}^3\rightarrow {\mathbb {C}}^3$, the magnetic flux density ${\mathbf {B}}:{\mathbb {R}}^3\rightarrow {\mathbb {C}}^3$, the magnetic field ${\mathbf {H}}:{\mathbb {R}}^3\rightarrow {\mathbb {C}}^3$, the current density ${\mathbf {J}}:{\mathbb {R}}^3\rightarrow {\mathbb {C}}^3$, and the known source current density ${\mathbf {J}}_0:{\mathbb {R}}^3\rightarrow {\mathbb {C}}^3$. These fields are the so-called Euclidean vector proxies [25, p. 243, Table 2.1] of the differential forms $u\in \Lambda ^{1}({\mathbb {R}}^3)$, $\varvec{\sigma }\in \Lambda ^{2}({\mathbb {R}}^3)$, ${\mathbf {j}}\in \Lambda ^{1}({\mathbb {R}}^3)$, and $\psi ,\psi _0\in \Lambda ^{2}({\mathbb {R}}^3)$ of Sect. 1.1.

The equilibrium equations (1.1a) become

1.2a $$\begin{aligned}&\left\{ \begin{array}{rcl} \nabla \times {\mathbf {A}}&{}=&{} {\mathbf {B}}{,}\\ \nabla \times {\mathbf {H}}&{}=&{} {\mathbf {J}}+ {\mathbf {J}}_0 {,}\end{array}\right. \end{aligned}$$

while the *constitutive equations* (*material laws*) read

1.2b $$\begin{aligned}&\left\{ \begin{array}{rcl} {\mathbf {H}}&{}=&{} \nu {\mathbf {B}}{,}\\ {\mathbf {J}}&{}=&{} -\imath \omega \sigma {\mathbf {A}}{,}\end{array}\right. \end{aligned}$$

where $\imath$ is the imaginary unit, $\nu ,\sigma \!:{\mathbb {R}}^3\rightarrow {\mathbb {R}}$ material parameters (reluctivity and conductivity), and $\omega \in {\mathbb {R}}$ the angular frequency. While $\omega$ is constant, $\nu ,\sigma \!:{\mathbb {R}}^3 \rightarrow {\mathbb {R}}$ generally vary in space. Note that multiplications with $\nu$ and $-\imath \omega \sigma$ correspond to the Hodge operators $\star _\alpha$ and $\star _\gamma$ in (1.1b).

#### Remark 2

According to [31, p. 259, Theorem 8.9] and [5], the appropriate decay condition for the eddy-current problem is

1.3 $$\begin{aligned} \Vert {\mathbf {A}}({\mathbf {x}}) \Vert = \mathcal {O}(\Vert {\mathbf {x}} \Vert ^{-1}) \quad \text {for } \Vert {\mathbf {x}} \Vert \rightarrow \infty \text { uniformly} {.}\end{aligned}$$

### Overview and outline

In this article, we present a *domain-decomposition approach* for the discretization of (1.1). We introduce a bounded domain $\Omega \subset {\mathbb {R}}^n$, $\Gamma :=\partial \Omega$, in whose complement $\Omega _{+}:={\mathbb {R}}^n \setminus \Omega$ we assume constant $\alpha ,\gamma \in {\mathbb {C}}$, that is $\star _\alpha$ and $\star _\gamma$ are induced by constant multiples of the Euclidean metric. Thus, we can view (1.1) as a transmission problem coupled across the interface $\Gamma$. Our main idea is to rely on different discretizations in $\Omega$ and $\Omega _{+}$:

- In $\Omega$, we want to employ a generic *mesh-based* discretization, which falls in the class of co-chain-based discretizations. This framework is the generalization of *Finite Element Exterior Calculus* (FEEC) [8] and *Discrete Exterior Calculus* (DEC) [22, 27, 38] and we elaborate it in Sect. 2.
- In the complement $\Omega _{+}$ of $\Omega$, filled with a homogeneous medium, we use a *Trefftz–Galerkin approximation*. It relies on basis functions that solve the homogeneous PDE, satisfy the right decay conditions, and span trial and test spaces to be plugged into variational formulations. Details will be given in Sect. 3.

The key issue is how to connect the different discretizations. Our proposals for this coupling represent the main novel contributions of this work. To develop our ideas, in Sect. 1.4 we examine different (variational) formulations of the transmission problem arising from (1.1).

Then, Sect. 4 discusses how to couple the discretizations across the interface $\Gamma$ separating $\Omega$ and $\Omega _{+}$. Starting from the natural transmission conditions for differential forms, we derive the fully-discrete coupled system of equations. This generalizes our earlier approaches to couple the *finite element method* with Trefftz methods: see [15] for Poisson’s equation, [16] for 2D Helmholtz equation, [17] for magnetostatic Maxwell’s equations, [19] for an eddy-current model as in Sect. 1.2, and [18] for electromagnetic wave propagation.

Finally, we return to the concrete eddy-current problem of Sect. 1.2 in Sects. 5 and 6. In Sect. 5, we show some examples of Trefftz basis functions and corresponding pure Trefftz blocks of the coupled systems. After that (Sect. 6), we also report simulation results for the concrete problem of Sect. 1.2 using the *cell method* (Sect. 6.1) for the mesh-based discretization.

The focus of this article is on the derivation and presentation of coupled discrete models for (1.1). It does not include a numerical analysis of convergence because rigorous theoretical results are even missing for some variants of the standalone cell method. Theoretical results are available in special cases of the finite element method and we refer the reader to the publications listed above.

### Variational formulations

We adopt the domain-decomposition setting introduced in Sect. 1.3: ${\mathbb {R}}^{n}=\Omega \cup \Gamma \cup \Omega _{+}$. For the sake of simplicity, we assume that the known source term $\psi _0\in \Lambda ^{m+1}({\mathbb {R}}^n)$ is supported inside $\Omega$. We elaborate two ways to arrive at a general transmission problem in ${\mathbb {R}}^n$ and establish related variational formulations.

#### Option I: primary formulation

Starting from (1.1), we eliminate all variables except for *u*, a procedure called *primary elimination* in [23, p. 276, (16)]:

1.4 $$\begin{aligned} \mathrm {d}\left( \star _\alpha \mathrm {d}u\right) = \left( -1\right) ^l\mathrm {d}\left( \star _\alpha \varvec{\sigma }\right) = \left( -1\right) ^l\mathrm {d}{\mathbf {j}}= \left( -1\right) ^l\left( \psi - \psi _0\right) = \left( -1\right) ^l\left( \star _\gamma u - \psi _0\right) \!{,}\end{aligned}$$

which can be rewritten as

1.5 $$\begin{aligned} \left( -1\right) ^{l-1}\mathrm {d}\left( \star _\alpha \mathrm {d}u\right) + \star _\gamma u = \psi _0 {.}\end{aligned}$$

For the derivation of the variational formulation, we focus on $\Omega$. Multiplication with $\eta \in \Lambda ^{l-1}(\Omega )$ and integration on $\Omega$ yields

1.6 $$\begin{aligned} \int \limits _\Omega \left[ \left( -1\right) ^{l-1}\mathrm {d}\left( \star _\alpha \mathrm {d}u\right) + \star _\gamma u\right] \wedge \eta = \int \limits _\Omega \psi _0 \wedge \eta \quad \forall \eta \in \Lambda ^{l-1}(\Omega ) {.}\end{aligned}$$

Integrating by parts [24, p. 254, (6)], we then obtain the (primary) weak formulation

1.7 $$\begin{aligned} \int \limits _\Omega \left[ \left( -1\right) ^n\left( \star _\alpha \mathrm {d}u \wedge \mathrm {d}\eta \right) + \star _\gamma u \wedge \eta \right] + \left( -1\right) ^{l-1}\int \limits _\Gamma \mathbf{t }\left( \star _\alpha \mathrm {d}u\right) \wedge \mathbf{t }\eta = \int \limits _\Omega \psi _0 \wedge \eta \quad \forall \eta \in \Lambda ^{l-1}(\Omega ) {,}\end{aligned}$$

where $\mathbf{t }:\Lambda ^{l}(\Omega )\rightarrow \Lambda ^{l}(\Gamma )$ is the (tangential, Dirichlet) trace of *l*-forms for any $l\in \left\{ 1,\dots ,n\right\}$ on $\Gamma$.

(1.7) forms the basis of the coupling approach for this problem (Sect. 4.1):

1. on the bounded domain $\Omega$, (1.7) is posed on a suitable Sobolev space of $\left( l-1\right)$-forms with square-integrable exterior derivative [8, 25], while
2. the influence of $\Omega _{+}$ is taken into account through the interface term of (1.7).

#### Option II: hybrid formulation

With the term “hybrid,” we refer to a *mixed* formulation involving a further potential for the exterior problem. We assume

- $\gamma = 0$ in $\Omega _{+}$, which implies $\left. \psi \right| _{\Omega _{+}}=0$ and $\left. \mathrm {d}{\mathbf {j}}\right| _{\Omega _{+}} = 0$ from (1.1a) and (1.1b), and that
- $\Omega _{+}$ has vanishing *m*-th Betti number^1^$\beta _m(\Omega _{+})=0$ [25, p. 246, Theorem 2.1].

We can then introduce a potential $\pi \in \Lambda ^{m-1}(\Omega _{+})$. The equations (1.1b) for the exterior problem become

$$\begin{aligned} \left\{ \begin{array}{ll} {\mathbf {j}}= \mathrm {d}\pi {,}\\ \mathrm {d}\left( \star _{\nicefrac {1}{\alpha }}\mathrm {d}\pi \right) = 0 {.}\\ \end{array}\right. \begin{array}{c} \quad \text {in }\Omega _{+}{.}\\ \end{array} \begin{array}{c} \qquad \qquad \qquad \qquad (1.8\mathrm{a})\\ \qquad \qquad \qquad \qquad (1.8\mathrm{b})\\ \end{array} \end{aligned}$$

To complete the problem, a suitable decay condition has to be imposed.

##### Remark 3

Throughout we rely on *ungauged* formulations even if $\gamma =0$ locally. The uniqueness of solution components $\varvec{\sigma }$ and ${\mathbf {j}}$ will not be affected.

The equation (1.1) for $\gamma =0$ allows to write $\star _\alpha \mathrm {d}u = \left( -1\right) ^{l}{\mathbf {j}}$ when restricted to $\Gamma$. Thus, we can recast the primary variational problem for $u \in \Lambda ^{l-1}(\Omega )$ and ${\mathbf {j}}\in \Lambda ^{m}(\Omega )$ as

1.9 $$\begin{aligned} \int \limits _{\Omega } \left[ \left( -1\right) ^n\left( \star _\alpha \mathrm {d}u\right) \wedge \mathrm {d}\eta + \star _\gamma u \wedge \eta \right] - \int \limits _{\Gamma } \mathbf{t }{\mathbf {j}}\wedge \mathbf{t }\eta = \int \limits _\Omega \psi _0 \wedge \eta \quad \forall \eta \in \Lambda ^{l-1}(\Omega ) {,}\end{aligned}$$

with ${\mathbf {j}}$ from the solution of (1.8).

##### Concrete Case

For the concrete case of the eddy-current problem presented in Sect. 1.2, the hybrid approach involves a magnetic scalar potential in $\Omega _{+}$. We assume that $\sigma = 0$ in $\Omega _{+}$, which implies $\left. {\mathbf {J}}\right| _{\Omega _{+}}=\mathbf {0}$ and $\left. \nabla \times {\mathbf {H}}\right| _{\Omega _{+}} = \mathbf {0}$ from (1.2a) and (1.2b). We then exploit that $\nabla \times {\mathbf {H}}= \mathbf {0}$ in $\Omega _{+}$ to switch to a potential representation there; $\Omega _{+}$ is supposed to have trivial topology, i.e., first Betti number^2^$\beta _1(\Omega _{+})=0$ [25, p. 252, Lemma 2.2]. This can be achieved by choosing $\Omega _{+}$ *simply connected*, if $\Omega$ has no handles.

We can then introduce a magnetic scalar potential $\phi \!:{\mathbb {R}}^3\rightarrow {\mathbb {C}}$ in $\Omega _{+}$. Hence, the eddy-current model in $\Omega _{+}$ is converted into

$$\begin{aligned} \left\{ \begin{array}{ll} {\mathbf {H}}= \nabla \phi {,}\\ \nabla \cdot \left( \nu _{+}^{-1}\nabla \phi \right) = 0 {,}\\ \end{array}\right. \begin{array}{c} \quad \text {in }\Omega _{+}{,}\\ \end{array} \begin{array}{c} \qquad \qquad \qquad \qquad (1.10\mathrm{a})\\ \qquad \qquad \qquad \qquad (1.10\mathrm{b})\\ \end{array} \end{aligned}$$

with $\nu _{+}=\nu$ in $\Omega _{+}$ and the decay condition

1.11 $$\begin{aligned} \phi ({\mathbf {x}}) = \mathcal {O}(\Vert {\mathbf {x}} \Vert ^{-1}) \quad \text {for } \Vert {\mathbf {x}} \Vert \rightarrow \infty \text { uniformly} {.}\end{aligned}$$

## Co-chain calculus

The framework of *co-chain calculus*, explained in [10, 23, 24, 42] and [12, p. 155, Section 16], allows for a unified treatment of a wide class of finite element and finite volume schemes, building on the foundation established by other works like [11]. This framework is the generalization of *Finite Element Exterior Calculus* (FEEC) [7, 8], *Discrete Exterior Calculus* (DEC) [22, 27, 38], and the *cell method* [20, 40].

The starting point of co-chain calculus is the linear stationary or time-harmonic elliptic boundary value problem in differential forms (1.1) on $\Omega$. We aim at discretizing those forms^3^ by means of a *co-chain* representation. To that end, we introduce

1. a *primary mesh* $\mathcal {M}$, with $\mathcal {F}_k$ set of *k*-dimensional facets of such mesh and $N_k :=\#\mathcal {F}_k$, and
2. a *secondary mesh* $\widetilde{\mathcal {M}}$, with $\widetilde{N}_k :=\#\widetilde{\mathcal {F}}_k$ (quantities linked to the secondary mesh are tagged by a tilde),

on the domain $\Omega$. Note that, despite their names, these meshes can be unrelated;^4^ we refer to [23, p. 271, Definition 2] for the definition of a mesh.

We then associate to every *k*-form occurring in (1.1) a *k*-co-chain in either $\mathcal {M}$ or $\widetilde{\mathcal {M}}$, i.e., a mapping $\mathcal {F}_k \rightarrow {\mathbb {R}}$ (or $\widetilde{\mathcal {F}}_k \rightarrow {\mathbb {R}}$). Thus, every *k*-co-chain can be identified with its *coefficient vector* $\in {\mathbb {C}}^{N_k}$ (or $\in {\mathbb {C}}^{\widetilde{N}_k}$), tagged with $\vec {\cdot }$ (or $\widetilde{\cdot }\,$) in the following. In other words, we adopt the following discrete models^5^ for the unknown differential forms of (1.1):

2.1a $$\begin{aligned} u\,\in \Lambda ^{l-1}(\Omega )&\;\Longrightarrow \,\,\, \left( l-1\right) \text {-co-chain on }\mathcal {M} \,\!\iff \, \vec {{\mathbf {u}}}\,\in {\mathbb {C}}^{N_{l-1}} {,}\end{aligned}$$

2.1b $$\begin{aligned} \varvec{\sigma }\,\in \Lambda ^{l}(\Omega )&\;\Longrightarrow \,\,\, l\text {-co-chain on }\mathcal {M} \,\!\iff \, \vec {\varvec{\sigma }}\,\in {\mathbb {C}}^{N_{l}} {,}\end{aligned}$$

2.1c $$\begin{aligned} {\mathbf {j}}\,\in \Lambda ^{m}(\Omega )&\;\Longrightarrow \,\,\, m\text {-co-chain on }\widetilde{\mathcal {M}} \,\!\iff \, \widetilde{{\mathbf {j}}}\,\in {\mathbb {C}}^{N_{m}} {,}\end{aligned}$$

2.1d $$\begin{aligned} \psi \,\in \Lambda ^{m+1}(\Omega )&\;\Longrightarrow \,\,\, \left( m+1\right) \text {-co-chain on }\widetilde{\mathcal {M}} \,\!\iff \, \widetilde{\varvec{\psi }}\,\in {\mathbb {C}}^{N_{m+1}} {.}\end{aligned}$$

The entries of each coefficient vector, for example $\vec {{\mathbf {u}}}$, can be regarded as an approximation of integrals of the corresponding form, $u\in \Lambda ^{l-1}(\Omega )$, over the respective ($\left( l-1\right)$-dimensional) facets of the (primary) mesh. These integrals serve as *degrees of freedom*.

The representation (2.1) has to be supplemented by introducing matrix representations for all linear operators of (1.1). For instance, the (primary/secondary) *exterior derivative* matrices ${\mathbf {D}}^{k-1} \in \left\{ -1,0,1\right\} ^{N_k, N_{k-1}}$ and $\widetilde{{\mathbf {D}}}^{k-1} \in \left\{ -1,0,1\right\} ^{\widetilde{N}_k, \widetilde{N}_{k-1}}$ are the incidence matrices between *oriented* *k*- and $\left( k-1\right)$-dimensional facets of the primary/secondary mesh, which allow to write algebraic equations linking primary and secondary co-chain-based vectors of degrees of freedom (2.1) through the *discrete* equilibrium equations

2.2a $$\begin{aligned}&\left\{ \begin{array}{rcl} {\mathbf {D}}^{l-1}\vec {{\mathbf {u}}} &{}=&{} \left( -1\right) ^l\vec {\varvec{\sigma }} {,}\\ \widetilde{{\mathbf {D}}}^{m}\widetilde{{\mathbf {j}}} &{}=&{} \widetilde{\varvec{\psi }} - \widetilde{\varvec{\psi }}_0 {.}\end{array}\right. \end{aligned}$$

The discrete constitutive equations can be written as

2.2b $$\begin{aligned}&\left\{ \begin{array}{lcl} \widetilde{{\mathbf {K}}}_{m}^{l}\widetilde{{\mathbf {j}}} &{}=&{} {\mathbf {M}}_\alpha ^l\vec {\varvec{\sigma }} {,}\\ \widetilde{{\mathbf {K}}}_{m+1}^{l-1}\widetilde{\varvec{\psi }} &{}=&{} {\mathbf {M}}_\gamma ^{l-1}\vec {{\mathbf {u}}} {,}\end{array}\right. \end{aligned}$$

where matrices ${\mathbf {M}}_\alpha ^l,{\mathbf {M}}_\gamma ^{l-1},\widetilde{{\mathbf {K}}}_{m}^{l},\widetilde{{\mathbf {K}}}_{m+1}^{l-1}$ have to fulfill the following three requirements [24, p. 254]:

1. *Mass* matrices ${\mathbf {M}}_\alpha ^l \in {\mathbb {C}}^{N_l, N_l}$ and ${\mathbf {M}}_\gamma ^{l-1} \in {\mathbb {C}}^{N_{l-1}, N_{l-1}}$ need to be invertible.^6^ They are the essential building blocks of discrete Hodge operators.^7^
2. The *pairing* matrices $\widetilde{{\mathbf {K}}}_{m}^{l} \in {\mathbb {R}}^{N_{l},\widetilde{N}_{m}}$ and $\widetilde{{\mathbf {K}}}_{m+1}^{l-1} \in {\mathbb {R}}^{N_{l-1},\widetilde{N}_{m+1}}$ are discrete representatives^8^ of the $\wedge$-product $\int _{\Omega } f \wedge g$ for $f\in \Lambda ^{l}(\Omega )$, $g\in \Lambda ^{m}(\Omega )$ and $f\in \Lambda ^{l-1}(\Omega )$, $g\in \Lambda ^{m+1}(\Omega )$, respectively. Pairing matrices need to fulfill the algebraic relationship [24, p. 254, (4)]
  2.3 $$\begin{aligned} \widetilde{{\mathbf {K}}}_{m}^{l} = \left( -1\right) ^{lm}\left( {\mathbf {K}}_{l}^{m}\right) ^{\mathsf {T}}\iff {\mathbf {K}}_{l}^{m} = \left( -1\right) ^{lm}\left( \widetilde{{\mathbf {K}}}_{m}^{l}\right) ^{\mathsf {T}}\end{aligned}$$
  for any $l\in \left\{ 1,\dots ,n\right\}$, $m :=n-l$ in ${\mathbb {R}}^{n}$, $n\in {\mathbb {N}}^*$.
3. The third requirement is a discrete counterpart of the integration by parts formula [24, p. 254, (6)]
  2.4a $$\begin{aligned} \int \limits _\Omega \mathrm {d}f \wedge g&+\, \left( -1\right) ^{l} \int \limits _\Omega f \wedge \mathrm {d}g = \int \limits _\Gamma f \wedge g \end{aligned}$$
  for all $f\in \Lambda ^{l-1}(\Omega )$, $g\in \Lambda ^{m}(\Omega )$, $l+m=n$, $n\in {\mathbb {N}}^*$, which corresponds to
  2.4b $$\begin{aligned} \left( {\mathbf {D}}^{l-1}\vec {{\mathbf {u}}}\right) ^{\mathsf {H}}\widetilde{{\mathbf {K}}}_{m}^{l}\widetilde{{\mathbf {v}}}&= \left( -1\right) ^{l}\vec {{\mathbf {u}}}^{\,{\mathsf {H}}}\,\widetilde{{\mathbf {K}}}_{m+1}^{l-1}\widetilde{{\mathbf {D}}}^{m}\widetilde{{\mathbf {v}}} + \left( {\mathbf {T}}_\Gamma ^{l-1}\vec {{\mathbf {u}}}\right) ^{\mathsf {H}}\widetilde{{\mathbf {K}}}_{m,\Gamma }^{l-1}\widetilde{{\mathbf {T}}}_\Gamma ^{m}\,\widetilde{{\mathbf {v}}} \end{aligned}$$

for all $\vec {{\mathbf {u}}}\in {\mathbb {C}}^{N_{l-1}}$, $\widetilde{{\mathbf {v}}}\in {\mathbb {C}}^{\widetilde{N}_{m}}$. The *trace* matrices ${\mathbf {T}}_{\Gamma }^{l-1} \in \left\{ 0,1\right\} ^{N_{l-1}^\text {bnd}, N_{l-1}}$ and $\widetilde{{\mathbf {T}}}_{\Gamma }^{m} \in \left\{ 0,1\right\} ^{\widetilde{N}_{m}^\text {bnd}, \widetilde{N}_{m}}$, with $N_{l-1}^{\text {bnd}},\widetilde{N}_{m}^{\text {bnd}}$ the numbers of $\left( l-1\right)$- and *m*-dimensional primary/secondary mesh facets $\subset \Gamma$, select the degrees of freedom on $\Gamma$, while the boundary pairing matrix $\widetilde{{\mathbf {K}}}_{m,\Gamma }^{l-1} \in {\mathbb {R}}^{N_{l-1}^\text {bnd},\widetilde{N}_{m}^\text {bnd}}$ is only defined on $\Gamma$.

Next we elaborate the discrete formulations on $\Omega$ for both ways of Sect. 1.4 to state the transmission problem of Sect. 1.1.

### Option I: discrete primary formulation

We can now deduce an expression for the discrete form of (1.7), also given in [23, p. 276, *Primary elimination*, (16)]:

2.5 $$\begin{aligned} \left[ \left( -1\right) ^n\left( {\mathbf {D}}^{l-1}\right) ^{\mathsf {T}}{\mathbf {M}}_\alpha ^l{\mathbf {D}}^{l-1} + {\mathbf {M}}_\gamma ^{l-1} + \left( -1\right) ^{l-1}\left( {\mathbf {T}}_\Gamma ^{l-1}\right) ^{\mathsf {T}}{\mathbf {M}}_{\beta ,\Gamma }^{l-1}{\mathbf {T}}_\Gamma ^{l-1} \right] \vec {{\mathbf {u}}} = \widetilde{{\mathbf {K}}}_{m+1}^{l-1}\widetilde{\varvec{\psi }}_0 {.}\end{aligned}$$

$\widetilde{\varvec{\psi }}_0 \in {\mathbb {C}}^{\widetilde{N}_{m+1}}$ is a known vector determined by integrals of the source $\left( m+1\right)$-form $\psi _0$ over $\left( m+1\right)$-dimensional facets of the secondary mesh. Thus, the secondary mesh only comes into play through the right-hand side of (2.5) and does not matter for the ultimate system matrix. Conversely, ${\mathbf {M}}_{\beta ,\Gamma }^{l-1} \in {\mathbb {C}}^{N_{l-1}^\text {bnd}, N_{l-1}^\text {bnd}}$ is an abstract *boundary-energy* term related to the Dirichlet-to-Neumann (DtN) operator. We clarify its meaning in Sect. 4.1.

### Option II: discrete hybrid formulation

We can also discretize the variational form (1.9) in the general framework of co-chain calculus:

2.6 $$\begin{aligned} \left[ \left( -1\right) ^n\left( {\mathbf {D}}^{l-1}\right) ^{\mathsf {T}}{\mathbf {M}}_\alpha ^l{\mathbf {D}}^{l-1} + {\mathbf {M}}_\gamma ^{l-1} \right] \vec {{\mathbf {u}}} - \left( {\mathbf {T}}_\Gamma ^{l-1}\right) ^{\mathsf {T}}\widetilde{{\mathbf {K}}}_{m,\Gamma }^{l-1}\widetilde{{\mathbf {T}}}_{\Gamma }^{m}\widetilde{{\mathbf {j}}} = \widetilde{{\mathbf {K}}}_{m+1}^{l-1}\widetilde{\varvec{\psi }}_0 {,}\end{aligned}$$

where $\widetilde{{\mathbf {j}}} \in {\mathbb {C}}^{\widetilde{N}_{m}}$ is the coefficient vector whose entries are related to integrals of ${\mathbf {j}}\in \Lambda ^{m}(\Omega )$ over *m*-dimensional facets of the secondary mesh. Indeed, similarly to the derivation of (1.9) from (1.7), we exploited that ${\mathbf {M}}_{\beta ,\Gamma }^{l-1}{\mathbf {T}}_\Gamma ^{l-1} \vec {{\mathbf {u}}} = \left( -1\right) ^{l}\widetilde{{\mathbf {K}}}_{m,\Gamma }^{l-1}\widetilde{{\mathbf {T}}}_{\Gamma }^{m}\widetilde{{\mathbf {j}}}$ (DtN-property, [23, p. 275, (14c)]).

## Trefftz methods

*Trefftz methods* seek to approximate the solution of constant-coefficient boundary value problems in (unbounded) domains by means of finitely-many global basis functions that solve the equations of the problem exactly and satisfy suitable conditions at infinity [26]. Hence, the main feature that characterizes a Trefftz method is its own discrete *Trefftz space*, and the functional form of the corresponding discrete basis functions leads to different types of Trefftz methods. These functions can be:

- *Plane waves* or *(generalized) harmonic polynomials*, which constitute the most common choice [26].
- *Fundamental solutions* of the PDE-operators, which yield Trefftz functions with a central singularity placed outside the domain of approximation [30].
- Related to the above, fields generated by point sources solving homogeneous equations;^9^ we then get the *method of auxiliary sources*^10^ [44].

In spite of this diversity, all Trefftz methods share a desirable feature and a drawback. The former is the exponential convergence of their approximation error when the unknown possesses an analytic extension beyond the Trefftz approximation domain.^11^ This is proven formally for 2D Poisson’s equation in [15, p. 3, Section 2.2] and with numerical evidence for 2D Helmholtz equation and Maxwell’s equations in [16] and [18], respectively.

The drawback is that, as exact solutions of a PDE are global functions, simple choices for a basis of a Trefftz space may be almost linearly dependent. Hence, Trefftz basis functions typically lead to ill-conditioned dense matrices. Stability may then be an issue.

To overcome the near-linear interdependence, Trefftz basis functions can be made orthogonal by a change of basis [6] or by choosing them orthogonal in the first place. However, the impact of ill-conditioning is limited due to the low number of degrees of freedom required for Trefftz methods, given their exponential convergence.

Related to the above is the need of heuristic rules to build the discrete Trefftz spaces when the unknown field is difficult to approximate: exponential convergence of Trefftz methods fails when, e.g., the field features singularities. Then, large Trefftz spaces may be needed and instability can have such a large impact that the numerical solution becomes useless.

Coupling a Trefftz method with a volume-mesh-based method can be a way to overcome this issue. As a matter of fact, by truncating the mesh at an *artificial boundary* that does not coincide with any physical discontinuity, Trefftz methods can be applied to a region where the unknown is sufficiently easy to approximate that the details of choosing the Trefftz space do not have much impact and exponential convergence is preserved.^12^

### Trefftz spaces for transmission problems

To capture the exterior problem (1.1) subject to a suitable condition at infinity (Option I, Sect. 1.4.1), we want to use functions that belong to the Trefftz space^13^

3.1a $$\begin{aligned}&\mathcal {T}^{l-1}(\Omega _{+}) :=\Big \{ v \in \Lambda ^{l-1}(\Omega _{+})\!:\;\; \left( -1\right) ^{l-1}\mathrm {d}\left( \star _\alpha \mathrm {d}v\right) + \star _\gamma v = 0, \;\; \alpha ,\gamma \in {\mathbb {C}}, \nonumber \\&\qquad v \text { smooth and satisfying a suitable condition at infinity} \Big \} {.}\end{aligned}$$

Conversely, the Trefftz space of functions that solve the simplified exterior problem (1.8) (Option II, Sect. 1.4.2) exactly is

3.1b $$\begin{aligned}&\mathcal {T}^{m-1}(\Omega _{+}) :=\Big \{ v \in \Lambda ^{m-1}(\Omega _{+})\!:\;\; \mathrm {d}\left( \star _{\nicefrac {1}{\alpha }}\mathrm {d}v\right) = 0, \;\; \alpha \in {\mathbb {C}}, \nonumber \\&\qquad \mathrm {d}\left( \star \,v\right) =0, \nonumber \\&\qquad v \text { smooth and satisfying a suitable condition at infinity} \Big \} {.}\end{aligned}$$

Note that the requirement $\mathrm {d}\left( \star \,v\right) =0$ is just a gauge condition^14^ to ensure that we recover a unique potential in $\Omega _{+}$.

## Coupling strategy

Given co-chain calculus inside $\Omega$ (Sect. 2) and a Trefftz method in the complement $\Omega _{+}$ (Sect. 3), we can now devise a general discretization of coupled problems in differential forms (1.1) for both transmission problems of Sect. 1.4.

We first derive the corresponding coupled systems in variational form in the continuous case. Afterwards, we rely on the co-chain calculus of Sect. 2 and the Trefftz spaces of Sect. 3 to accomplish discretization.

Our approach weakly imposes transmission conditions across $\Gamma$. In a sense, it is inspired by the so-called *three-field variational formulation* proposed for the sake of domain decomposition in [13]. Owing to the Trefftz approximation, our coupling approach can also be regarded as a generalization of the DtN-based coupling from [15, p. 1519, Section 3.2], [19, p. 3, Section V.A], and [18, p. 13, Section 3.3.2], where it is proposed in a finite-element context.

### Option I: primary formulation

Transmission conditions are required between $\Omega$ and $\Omega _{+}$ [32, p. 107, Lemma 5.3]:

$$\begin{aligned} \left\{ \begin{array}{cc} \mathbf{t } \Big (\left. \star _\alpha \mathrm {d} u\right| _{\Omega }\Big ) =\, \mathbf{t } \Big (\left. \star _\alpha \mathrm {d} u\right| _{\Omega _{+}}\Big ) \\ \mathbf{t } \left. u\right| _{\Omega } =\, \mathbf{t } \left. u\right| _{\Omega _{+}}\\ \end{array}\right. \begin{array}{c} \quad \text {on }\Gamma {.}\\ \end{array} \begin{array}{c} \qquad \qquad \qquad \qquad (4.1\mathrm{a})\\ \qquad \qquad \qquad \qquad (4.1\mathrm{b})\\ \end{array} \end{aligned}$$

On one hand, we plug (4.1a) into (1.7) directly. On the other hand, we impose (4.1b) weakly using as test functions the so-called *co-normal traces* $\mathbf{t } \left( -1\right) ^l\star _\alpha \mathrm {d}v$ of Trefftz functions $v \in \mathcal {T}^{l-1}(\Omega _{+})$ of (3.1a). This yields the coupled variational problem

4.2 $$\begin{aligned} \boxed { \begin{aligned}&\text {Seek} \;\; u \in \Lambda ^{l-1}(\Omega ),\, \,\,v \in \mathcal {T}^{l-1}(\Omega _{+})\!: \\&\begin{array}{rcccl} \int \limits _{\Omega } \left[ \left( -1\right) ^n\left( \star _\alpha \mathrm {d}u \wedge \mathrm {d}\eta \right) + \star _\gamma u \wedge \eta \right] + \left( -1\right) ^{l-1}\int \limits _{\Gamma } \mathbf{t }\left( \star _\alpha \mathrm {d}v\right) \wedge \mathbf{t }\eta &{} = \int \limits _\Omega \psi _0 \wedge \eta \\ \left( -1\right) ^{l-1}\int \limits _{\Gamma } \mathbf{t }u \wedge \mathbf{t }\left( \star _\alpha \mathrm {d}\zeta \right) - \left( -1\right) ^{l-1}\int \limits _{\Gamma } \mathbf{t }v \wedge \mathbf{t }\left( \star _\alpha \mathrm {d}\zeta \right) &{}= 0 \end{array} \\&\,\,\! \forall \eta \in \Lambda ^{l-1}(\Omega ),\, \forall \zeta \in \mathcal {T}^{l-1}(\Omega _{+}) {.}\end{aligned}} \end{aligned}$$

Another expression for (4.2) can be obtained by noticing that

4.3 $$\begin{aligned} \left( -1\right) ^{l}\int \limits _{\Gamma } \mathbf{t }\left( \star _\alpha \mathrm {d}v\right) \wedge \mathbf{t }\zeta = \int \limits _{\Omega _{+}} \left[ \left( -1\right) ^n\left( \star _\alpha \mathrm {d}v \wedge \mathrm {d}\zeta \right) + \star _\gamma v \wedge \zeta \right] {,}\end{aligned}$$

which holds by integration by parts because of $v \in \mathcal {T}^{l-1}(\Omega _{+})$. We then obtain the coupled problem in variational form

4.4 $$\begin{aligned} \boxed { \begin{aligned}&\text {Seek} \;\; u \in \Lambda ^{l-1}(\Omega ),\, \,\,v \in \mathcal {T}^{l-1}(\Omega _{+})\!: \\&\begin{array}{rcccl} \int \limits _{\Omega } \left[ \left( -1\right) ^n\left( \star _\alpha \mathrm {d}u \wedge \mathrm {d}\eta \right) + \star _\gamma u \wedge \eta \right] + \left( -1\right) ^{l-1}\int \limits _{\Gamma } \mathbf{t }\left( \star _\alpha \mathrm {d}v\right) \wedge \mathbf{t }\eta &{} = \int \limits _\Omega \psi _0 \wedge \eta \\ \left( -1\right) ^{l-1}\int \limits _{\Gamma } \mathbf{t }u \wedge \mathbf{t }\left( \star _\alpha \mathrm {d}\zeta \right) + \int \limits _{\Omega _{+}} \left[ \left( -1\right) ^n\left( \star _\alpha \mathrm {d}v \wedge \mathrm {d}\zeta \right) + \star _\gamma v \wedge \zeta \right] &{}= 0 \end{array} \\&\,\,\! \forall \eta \in \Lambda ^{l-1}(\Omega ),\, \forall \zeta \in \mathcal {T}^{l-1}(\Omega _{+}) {.}\end{aligned}} \end{aligned}$$

The bottom-right integral in (4.4) expresses the “energy” associated with (1.5) in $\Omega _{+}$.

**Discretization** Similarly to the discrete counterpart (2.5) of the weak formulation (1.7), we can write the discrete version of (4.2) and (4.4) in abstract algebraic form

4.5 $$\begin{aligned} \boxed { \begin{array}{rcccl} \left[ \left( -1\right) ^n({\mathbf {D}}^{l-1})^{\mathsf {T}}{\mathbf {M}}_\alpha ^l{\mathbf {D}}^{l-1} + {\mathbf {M}}_\gamma ^{l-1}\right] \vec {{\mathbf {u}}} + ({\mathbf {T}}_\Gamma ^{l-1})^{\mathsf {T}}\widetilde{{\mathbf {K}}}_{m,\Gamma }^{l-1}\widetilde{{\mathbf {P}}}_{\Gamma } \vec {{\mathbf {v}}} &{}=&{} \widetilde{{\mathbf {K}}}_{m+1}^{l-1} \widetilde{\varvec{\psi }}_0 \\ \widetilde{{\mathbf {P}}}_{\Gamma }^{\,{\mathsf {H}}}(\widetilde{{\mathbf {K}}}_{m,\Gamma }^{l-1})^{\mathsf {T}}{\mathbf {T}}_\Gamma ^{l-1} \vec {{\mathbf {u}}} + {\mathbf {M}}_{+} \vec {{\mathbf {v}}} &{}=&{} \mathbf{0} \end{array}} \end{aligned}$$

using, on top of the terms of (2.5), the other following terms:

- We call $\widetilde{{\mathbf {P}}}_{\Gamma } \in {\mathbb {C}}^{\widetilde{N}_m^\text {bnd}, N_{+}}$ *co-normal trace projection* matrix, with $N_{+}$ dimension of the discrete Trefftz space $\mathcal {T}^{l-1}_h(\Omega _{+}) \subset \mathcal {T}^{l-1}(\Omega _{+})$. Comparing (4.2) and (4.5), it is clear that $\widetilde{{\mathbf {P}}}_{\Gamma }$ is the matrix representation of a mapping $\mathcal {T}^{l-1}(\Omega _{+}) \rightarrow \left\{ m\text {-co-chain on }\!\left. \widetilde{\mathcal {M}}\right| _{\Gamma }\right\}$.
- $\vec {{\mathbf {v}}} \in {\mathbb {C}}^{N_{+}}$ is the vector of coefficients with respect to a basis of the discrete Trefftz space $\mathcal {T}^{l-1}_h(\Omega _{+})$.
- ${\mathbf {M}}_{+} \in {\mathbb {C}}^{N_{+}, N_{+}}$ is the *energy* matrix in $\Omega _{+}$:
  4.6 $$\begin{aligned} \left( {\mathbf {M}}_{+}\right) _{i,j} :=\int \limits _{\Omega _{+}} \left[ \left( -1\right) ^n\left( \star _\alpha \mathrm {d}v_i \wedge \mathrm {d}v_j\right) + \star _\gamma v_i \wedge v_j\right] {,}\end{aligned}$$
  with $\left\{ v_k\right\}$, $k=1,\dots ,N_{+}$, a basis of $\mathcal {T}^{l-1}_h(\Omega _{+})$.

To arrive at an alternative expression of (4.5) involving ${\mathbf {M}}_{+}$, we note that the total number of degrees of freedom of the Trefftz discretization, $N_{+}$, is generally low because, under certain conditions, Trefftz methods enjoy exponential convergence (see Sect. 3). Thus, ${\mathbf {M}}_{+}$ can easily be inverted by Gaussian elimination, and we can write the Schur complement of (4.5):

4.7 $$\begin{aligned} \boxed { \begin{aligned} \left[ \left( -1\right) ^n({\mathbf {D}}^{l-1})^{\mathsf {T}}{\mathbf {M}}_\alpha ^l{\mathbf {D}}^{l-1} + {\mathbf {M}}_\gamma ^{l-1} - \left( {\mathbf {T}}_\Gamma ^{l-1}\right) ^{\mathsf {T}}\widetilde{{\mathbf {K}}}_{m,\Gamma }^{l-1}\widetilde{{\mathbf {P}}}_{\Gamma }{\mathbf {M}}_{+}^{-1}\widetilde{{\mathbf {P}}}_{\Gamma }^{\,{\mathsf {H}}}(\widetilde{{\mathbf {K}}}_{m,\Gamma }^{l-1})^{\mathsf {T}}{\mathbf {T}}_\Gamma ^{l-1}\right] \vec {{\mathbf {u}}}&= \\ \widetilde{{\mathbf {K}}}_{m+1}^{l-1} \widetilde{\varvec{\psi }}_0&{.}\end{aligned}} \end{aligned}$$

We can now compare the left-hand side of (4.7) with the original discretization (2.5) and write

4.8 $$\begin{aligned} \left( -1\right) ^{l}{\mathbf {M}}_{\beta ,\Gamma }^{l-1} \equiv \widetilde{{\mathbf {K}}}_{m,\Gamma }^{l-1}\widetilde{{\mathbf {P}}}_{\Gamma }{\mathbf {M}}_{+}^{-1}\widetilde{{\mathbf {P}}}_{\Gamma }^{\,{\mathsf {H}}}(\widetilde{{\mathbf {K}}}_{m,\Gamma }^{l-1})^{\mathsf {T}}\!{.}\end{aligned}$$

In this way, ${\mathbf {M}}_{+}$ provides a concrete expression for ${\mathbf {M}}_{\beta ,\Gamma }^{l-1}$. Note that (4.8) is also the discrete version of the integration by parts formula (4.3).

### Option II: hybrid formulation

For the hybrid problem (1.1) and (1.8), transmission conditions between $\Omega$ and $\Omega _{+}$ are

$$\begin{aligned} \left\{ \begin{array}{ll} \mathbf{t } \left. {\mathbf {j}}\right| _{\Omega } ~~=\, \mathbf{t } \Big (\left. \mathrm {d}\pi \right| _{\Omega _{+}}\Big ) \\ \mathbf{t } \left. \varvec{\sigma }\right| _{\Omega } =\, \mathbf{t }\Big (\left. \star _{\nicefrac {1}{\alpha }}\mathrm {d}\pi \right| _{\Omega _{+}}\Big )\\ \end{array}\right. \begin{array}{c} \quad \text {on }\Gamma {.}\\ \end{array} \begin{array}{c} \qquad \qquad \qquad \qquad (4.9\mathrm{a})\\ \qquad \qquad \qquad \qquad (4.9\mathrm{b})\\ \end{array} \end{aligned}$$

To impose these conditions, similarly to what was done for (1.9), we can write that $\star _\alpha \mathrm {d}v = \left( -1\right) ^{l}{\mathbf {j}}$ in $\Omega _{+}$. (4.4) (with $\gamma =0$ in $\Omega _{+}$) can therefore be rewritten as

4.10 $$\begin{aligned} \boxed { \begin{aligned}&\text {Seek} \;\; u \in \Lambda ^{l-1}(\Omega ),\, \,\,{\mathbf {j}}\in \mathcal {T}^{m}(\Omega _{+})\!: \\&\int \limits _{\Omega } \left[ \left( -1\right) ^n\left( \star _\alpha \mathrm {d}u \wedge \mathrm {d}\eta \right) + \star _\gamma u \wedge \eta \right] - \int \limits _{\Gamma } \mathbf{t }{\mathbf {j}}\wedge \mathbf{t }\eta = \int \limits _\Omega \psi _0 \wedge \eta \\&\quad -\int \limits _{\Gamma } \mathbf{t }u \wedge \mathbf{t }\varvec{\iota } + \left( -1\right) ^n\int \limits _{\Omega _{+}} {\mathbf {j}}\wedge \star _{\nicefrac {1}{\alpha }}\varvec{\iota } = 0 \\&\qquad \,\,\! \forall \eta \in \Lambda ^{l-1}(\Omega ),\, \forall \varvec{\iota } \in \mathcal {T}^{m}(\Omega _{+}) {.}\end{aligned}} \end{aligned}$$

Note that ${\mathbf {j}},\varvec{\iota }$ belong to the Trefftz space $\mathcal {T}^{m}(\Omega _{+})$, which correspond to $\mathcal {T}^{l-1}(\Omega _{+})$ of (3.1a) after taking co-normal traces $\mathbf{t } \left( -1\right) ^l\star _\alpha \mathrm {d}v$ of its functions *v*.

Let us now take $\pi ,\tau \in \mathcal {T}^{m-1}(\Omega _{+})$ of (3.1b) such that, in $\Omega _{+}$, ${\mathbf {j}}= \mathrm {d}\pi$ and $\varvec{\iota } = \mathrm {d}\tau$. System (4.10) then becomes^15^

4.11 $$\begin{aligned} \boxed { \begin{aligned}&\text {Seek} \;\; u \in \Lambda ^{l-1}(\Omega ),\, \,\,\pi \in \mathcal {T}^{m-1}(\Omega _{+})\!: \\&\int \limits _{\Omega } \left[ \left( -1\right) ^n\left( \star _\alpha \mathrm {d}u \wedge \mathrm {d}\eta \right) + \star _\gamma u \wedge \eta \right] - \int \limits _{\Gamma } \mathbf{t }\left( \mathrm {d}\pi \right) \wedge \mathbf{t }\eta = \int \limits _\Omega \psi _0 \wedge \eta \\&\qquad -\int \limits _{\Gamma } \mathbf{t }u \wedge \mathbf{t }\left( \mathrm {d}\tau \right) + \left( -1\right) ^n\int \limits _{\Omega _{+}} \mathrm {d}\pi \wedge \star _{\nicefrac {1}{\alpha }}\mathrm {d}\tau = 0 \\&\qquad \! \forall \eta \in \Lambda ^{l-1}(\Omega ),\, \forall \tau \in \mathcal {T}^{m-1}(\Omega _{+}) \end{aligned}} \end{aligned}$$

for the Trefftz space $\mathcal {T}^{m-1}(\Omega _{+})$ defined in (3.1b).

In (4.11), we can finally replace the integral in $\Omega _{+}$ with an integral on $\Gamma$, similarly to (4.3).

**Discretization** Similarly to (4.4) and (4.5), the variational formulation (4.11) can be linked to a linear system of equations in a discrete setting:

4.12 $$\begin{aligned} \boxed { \begin{array}{rcccl} \left[ \left( -1\right) ^n({\mathbf {D}}^{l-1})^{\mathsf {T}}{\mathbf {M}}_\alpha ^l{\mathbf {D}}^{l-1} + {\mathbf {M}}_\gamma ^{l-1}\right] \vec {{\mathbf {u}}} + ({\mathbf {T}}_\Gamma ^{l-1})^{\mathsf {T}}\widetilde{{\mathbf {K}}}_{m,\Gamma }^{l-1}\widetilde{{\mathbf {D}}}_{\Gamma }^{m-1}\widetilde{{\mathbf {P}}}_{\Gamma } \vec {{\mathbf {v}}} &{}= \widetilde{{\mathbf {K}}}_{m+1}^{l-1} \widetilde{\varvec{\psi }}_0 \\ \widetilde{{\mathbf {P}}}_{\Gamma }^{\,{\mathsf {H}}}(\widetilde{{\mathbf {D}}}_{\Gamma }^{m-1})^{\mathsf {T}}(\widetilde{{\mathbf {K}}}_{m,\Gamma }^{l-1})^{\mathsf {T}}{\mathbf {T}}_\Gamma ^{l-1} \vec {{\mathbf {u}}} + {\mathbf {M}}_{+} \vec {{\mathbf {v}}} &{} = \mathbf{0} \end{array}} \end{aligned}$$

Note the occurrence of an additional boundary incidence matrix, $\widetilde{{\mathbf {D}}}_{\Gamma }^{m-1} \in \left\{ -1,0,1\right\} ^{\widetilde{N}_{m}^\text {bnd}, \widetilde{N}_{m-1}^\text {bnd}}$. Also note that, abusing the notation of (4.5), we kept the symbols $\widetilde{{\mathbf {P}}}_{\Gamma } \in {\mathbb {C}}^{\widetilde{N}_{m-1}^\text {bnd}, N_{+}}$ and ${\mathbf {M}}_{+} \in {\mathbb {C}}^{N_{+}, N_{+}}$ for the co-normal trace projection matrix and the energy matrix, even though here they have a different construction (and $\widetilde{{\mathbf {P}}}_{\Gamma } \in {\mathbb {C}}^{\widetilde{N}_{m}^\text {bnd}, N_{+}}$ in (4.5)).

The next section will provide some details on the linear systems (4.5) and (4.12) for the specific case of the eddy-current equations from Sect. 1.2.

## Specialization

Here, for the eddy-current problem introduced in Sect. 1.2, we present

- transmission conditions across $\Gamma$, which separates the co-chain-based and Trefftz discretizations,
- a basis for the Trefftz spaces of Sect. 3, and
- the construction of the matrix of Sect. 4 involving only^16^ Trefftz degrees of freedom, i.e., ${\mathbf {M}}_{+}$.

### Option I: discrete primary formulation

Analogously to (4.1), the appropriate transmission conditions across $\Gamma$ are the tangential continuities of ${\mathbf {A}}$ and $\nu \,\nabla \times {\mathbf {A}}$. For the sake of simplicity, we also assume constant $\nu =\nu _{+}>0$ and $\sigma =\sigma _{+}>0$ in $\Omega _{+}$.

Hence, given a spherical coordinate system in ${\mathbb {R}}^3$ ($r\in [0,\infty )$, $\theta \in [0,2\pi )$, $\varphi \in [0,\pi ]$) with respect to a center ${\mathbf {c}}\in {\mathbb {R}}^3$ (${\mathbf {c}}$ being a position vector in Cartesian coordinates), Trefftz basis functions in the discrete version $\mathcal {T}^{1}_h(\Omega _{+})$ of (3.1a) satisfying the equations (1.2) and (1.3) in $\Omega _{+}$ can have the form^17^

5.1 $$\begin{aligned} \left( r,\theta ,\varphi \right) \mapsto \left\{ \begin{aligned}&h_{\mathsf {l}}^{(1)}(\kappa r_{xc})\,\varvec{\Phi }_{\mathsf {l}\mathsf {m}}(\theta _{xc},\varphi _{xc}) {,}\\&\mathsf {l}(\mathsf {l}+1)\,\frac{h_{\mathsf {l}}^{(1)}(\kappa r_{xc})}{\kappa r_{xc}}\,{\mathbf {Y}}_{\mathsf {l}\mathsf {m}}(\theta _{xc},\varphi _{xc}) + \left[ h_{\mathsf {l}}^{(1)\,\prime }(\kappa r_{xc}) + \frac{h_{\mathsf {l}}^{(1)}(\kappa r_{xc})}{\kappa r_{xc}}\right] \varvec{\Psi }_{\mathsf {l}\mathsf {m}}(\theta _{xc},\varphi _{xc}) {,}\\&\mathsf {l} = 1,\dots ,\infty , \quad \mathsf {m} = -\mathsf {l},\dots ,\mathsf {l}, \end{aligned} \right. \end{aligned}$$

where $\left( r_{xc},\theta _{xc},\varphi _{xc}\right) ^\top$ are the spherical coordinates of the vector ${\mathbf {x}}_c :={\mathbf {x}}- {\mathbf {c}}$ (${\mathbf {x}}$ another Cartesian position vector). *Vector spherical harmonics* [14, p. 289] are defined as

5.2a $$\begin{aligned} {\mathbf {Y}}_{\mathsf {l}\mathsf {m}}(\theta ,\varphi )&:=\; {\mathbf {e}}_r\, Y_{\mathsf {l}\mathsf {m}}(\theta ,\varphi ), \quad {\mathbf {e}}_r=\left( 1,0,0\right) ^{\mathsf {T}}\!, \end{aligned}$$

5.2b $$\begin{aligned} \varvec{\Phi }_{\mathsf {l}\mathsf {m}}(\theta ,\varphi )&:=\; {\mathbf {r}}\times \nabla _\text {sph} Y_{\mathsf {l}\mathsf {m}}(\theta ,\varphi ), \quad {\mathbf {r}}=\left( r,0,0\right) ^{\mathsf {T}}\!, \end{aligned}$$

5.2c $$\begin{aligned} \varvec{\Psi }_{\mathsf {l}\mathsf {m}}(\theta ,\varphi )&:=\; r\, \nabla _\text {sph} Y_{\mathsf {l}\mathsf {m}}(\theta ,\varphi ), \end{aligned}$$

here with spherical components. Moreover,

- $h_{\mathsf {l}}^{(1)}$ is a spherical Hankel function of the first kind [31, p. 281],
- $\kappa :=\sqrt{\frac{\omega \sigma _{+}}{\imath \;\nu _{+}}} \in {\mathbb {C}}$ is a constant parameter in $\Omega _{+}$, and
- $\nabla _\text {sph}$ denotes the gradient in spherical coordinates and $Y_{\mathsf {l}\mathsf {m}}(\theta ,\varphi )$ the spherical harmonics [28, p. 108, (3.53)]. It can be shown that $\varvec{\Phi }_{\mathsf {l}\mathsf {m}},\varvec{\Psi }_{\mathsf {l}\mathsf {m}}$ do not depend on *r* despite its presence in their definitions (5.2b) and (5.2c).

Finally, ${\mathbf {M}}_{+}$ of (4.5) has the following entries:

5.3 $$\begin{aligned} \left( {\mathbf {M}}_{+}\right) _{i,j} :=-\int \limits _{\Omega _{+}} \left[ \nu _{+} \left( \nabla \times {\mathbf {v}}_i\right) \cdot \left( \nabla \times {\mathbf {v}}_j\right) + \imath \omega \sigma _{+} {\mathbf {v}}_i\cdot {\mathbf {v}}_j \right] \mathrm {d}{\mathbf {x}}= \nu _{+}\int \limits _{\Gamma } \left[ {\mathbf {n}}\times \left( \nabla \times {\mathbf {v}}_i\right) \right] \cdot {\mathbf {v}}_j \;\mathrm {d}S{,}\end{aligned}$$

where ${\mathbf {v}}_j$, $j=1,\dots ,N_{+}$, are the Trefftz basis functions (5.1) of $\mathcal {T}^{1}_h(\Omega _{+})$, ${\mathbf {n}}$ the normal vector on $\Gamma$, and the second equality holds because of Trefftz functions in $\mathcal {T}^{1}(\Omega _{+})$ being exact solutions of the homogeneous problem and integration by parts.

### Option II: discrete hybrid formulation

Now as transmission conditions across $\Gamma$ we have to impose matching tangential components of $\nu \,\nabla \times {\mathbf {A}}$ and $\nabla \phi$, and matching normal components of $\nabla \times {\mathbf {A}}$ and $\nu _{+}^{-1}\nabla \phi$ (see (4.9)). For the sake of simplicity, we also assume constant $\nu =\nu _{+}>0$ in $\Omega _{+}$.

To discretize the Trefftz space $\mathcal {T}^{0}(\Omega _{+})$ of (3.1b), we can then choose the Trefftz basis functions

5.4 $$\begin{aligned} \left( r,\theta ,\varphi \right) \mapsto r_{xc}^{-\mathsf {l}-1}Y_{\mathsf {l}\mathsf {m}}(\theta _{xc},\varphi _{xc}), \quad \mathsf {l} = 0,\dots ,\infty , \quad \mathsf {m} = -\mathsf {l},\dots ,\mathsf {l} {.}\end{aligned}$$

Hence, for the special transmission problem (1.10) and (1.11) in $\Omega _{+}$, the entries of ${\mathbf {M}}_{+}$ in (5.3) are

5.5 $$\begin{aligned} \left( {\mathbf {M}}_{+}\right) _{i,j} :=-\nu _{+}^{-1}\int \limits _{\Omega _{+}} \nabla v_i \cdot \nabla v_j \;\mathrm {d}{\mathbf {x}}= -\nu _{+}^{-1}\int \limits _{\Gamma } \left( {\mathbf {n}}\cdot \nabla v_i\right) \, v_j \;\mathrm {d}S{.}\end{aligned}$$

$v_j$, $j=1,\dots ,N_{+}$, are the chosen Trefftz basis functions (5.4) in $\mathcal {T}^{0}_h(\Omega _{+})$ and the second equality in (5.5) again holds because of Trefftz functions in $\mathcal {T}^{0}(\Omega _{+})$ being exact solutions of the homogeneous problem.

## Numerical simulation

We again consider the eddy-current problem (1.2) in $\Omega$ and (1.10) in $\Omega _{+}$ with the radiation condition (1.11). This problem is solved for the magnetic vector potential ${\mathbf {A}}$ in $\Omega$ using the cell method (Sect. 6.1) as co-chain-based discretization and the magnetic scalar potential $\phi$ in $\Omega _{+}$ using Trefftz functions (5.4), hence by the coupling based on the hybrid formulation as discussed in Sect. 4.2 (Option II), which is specialized for the case of the cell method in Sect. 6.2.

### Cell method

The Cell Method (from now on, CM), established in [40], relies on a pair of meshes for the spatial discretization of boundary value problems: one mesh being the *dual* of the other. Specimens of such dual mesh can be built by a Delaunay–Voronoi subdivision, as proposed in [41], whereas barycentric dual meshes are used in [4, 20, 33, 34] for computational advantages [40, p. 13].

CM is an example of a method that can be fitted into the co-chain-based framework presented in Sect. 2. In particular, CM degrees of freedom are integrals of fields on entities of the primary and dual meshes.^18^ In the context of electromagnetics, where CM has long been used, examples are fluxes of the magnetic flux density on primary faces or line integrals of the magnetic field on dual edges [41]. This implies^19^ that the CM discretization, as a particular case of co-chain calculus, replaces differential operators with incidence matrices. In this way, equilibrium equations involving such operators (e.g., (1.2a)) can be enforced exactly.

Basis functions are then required to interpolate fields locally in terms of these integral degrees of freedom and approximate the material laws (constitutive equations, like (1.2b)). With the choice of Whitney basis functions, the matrices of FEEC can be recovered [40, 41].

Vector fields can also be interpolated by the piecewise-constant vector basis functions defined in [21] for *tetrahedral* cells. Specifically, positive-definite mass matrices in the CM discretization scheme can be obtained, for instance, by the so-called *energy approach* described in [21, 36], which makes it possible to exactly reconstruct the energy in a domain $\Omega$ with a piecewise-constant field.

Here we give examples of such mass matrices for the eddy-current model of Sect. 1.2 (which corresponds to the case $n=3$, $l=2$, $m=1$). We recognize the general parameters $\alpha$ and $\gamma$ as $\nu$ (reluctivity) and $-\imath \omega \sigma$ ($\sigma$ is conductivity), respectively, so that the general mass matrices of Sect. 2, ${\mathbf {M}}^2_\alpha$ and ${\mathbf {M}}^1_\gamma$ become ${\mathbf {M}}_\nu$ and $-\imath \omega {\mathbf {M}}_\sigma$, where ${\mathbf {M}}_{\nu } \in {\mathbb {R}}^{N_\text {faces}, N_\text {faces}}$ and ${\mathbf {M}}_{\sigma } \in {\mathbb {R}}^{N_\text {edges}, N_\text {edges}}$ (given $N_\text {faces}$ number of faces and $N_\text {edges}$ number of edges of the primary mesh $\mathcal {M}$) are defined as:

6.1a $$\begin{aligned} \left( {\mathbf {M}}_{\nu }\right) _{i,j}&= \int \limits _{\Omega } \nu ({\mathbf {x}})\, {\mathbf {b}}_i^\mathrm {f}({\mathbf {x}})\cdot {\mathbf {b}}_j^\mathrm {f}({\mathbf {x}}) \,\mathrm {d}{\mathbf {x}}\end{aligned}$$

and

6.1b $$\begin{aligned} \left( {\mathbf {M}}_{\sigma }\right) _{i,j}&= \int \limits _{\Omega } \sigma ({\mathbf {x}})\, {\mathbf {b}}_i^\mathrm {e}({\mathbf {x}})\cdot {\mathbf {b}}_j^\mathrm {e}({\mathbf {x}}) \,\mathrm {d}{\mathbf {x}}. \end{aligned}$$

Here, ${\mathbf {b}}_i^\mathrm {f} \in {\mathbf {L}}^2(\Omega )$ is the *vector face basis function* related to the *i*-th face of the *tetrahedral* mesh $\mathcal {M}$ and ${\mathbf {b}}_j^\mathrm {e} \in {\mathbf {L}}^2(\Omega )$ is the *vector edge basis function* related to the *j*-th edge of $\mathcal {M}$.

### CM–Trefftz coupling

For the eddy-current model introduced in Sect. 1.2, assuming constant $\nu =\nu _{+}>0$ in $\Omega _{+}$, the projection matrix $\widetilde{{\mathbf {P}}}_{\Gamma } \in {\mathbb {C}}^{\widetilde{N}_\text {edges}^\text {bnd}, N_{+}}$ (linking the CM and Trefftz discretizations) can be constructed as

6.2a $$\begin{aligned} \left( \widetilde{{\mathbf {P}}}_{\Gamma }\right) _{i,j}&= -\nu _{+} \int \limits _{\widetilde{\ell }_i} \left[ {\mathbf {n}}\times \left( \nabla \times {\mathbf {v}}_j\right) \right] \cdot \mathrm {d}\vec {{\varvec{s}}}\end{aligned}$$

for ${\mathbf {v}}_j$, $j=1,\dots ,N_{+}$, chosen Trefftz basis functions of $\mathcal {T}^{1}_h(\Omega _{+})$, like (5.1) (Option I), where $\widetilde{\ell }_i$, $i=1,\dots ,\widetilde{N}_\text {edges}^\text {bnd}$, is an edge of $\widetilde{\mathcal {M}}$ on $\Gamma$ and ${\mathbf {n}}$ its normal vector. Conversely, for $v_j$ chosen Trefftz basis functions of $\mathcal {T}^{0}_h(\Omega _{+})$, like (5.4) (Option II), the entries of $\widetilde{{\mathbf {P}}}_{\Gamma }$ are

6.2b $$\begin{aligned} \left( \widetilde{{\mathbf {P}}}_{\Gamma }\right) _{i,j}&= \nu _{+} \int \limits _{\widetilde{\ell }_i} \varvec{\tau } \cdot \nabla v_j \;\mathrm {d}s = \nu _{+}\, \left[ \epsilon _{i,1} v_j(\widetilde{{\mathbf {x}}}_{i,1}) + \epsilon _{i,2} v_j(\widetilde{{\mathbf {x}}}_{i,2})\right] {,}\end{aligned}$$

with $\varvec{\tau }$ the unit tangential vector of $\widetilde{\ell }_i$, $\widetilde{{\mathbf {x}}}_{i,1},\widetilde{{\mathbf {x}}}_{i,2}$ the endpoints of $\widetilde{\ell }_i$ (which correspond to centroids of faces of $\mathcal {M}$ on $\Gamma$, using a barycentric dual mesh as $\widetilde{\mathcal {M}}$), and $\epsilon _{i,1},\epsilon _{i,2}$ being $=1$ or $-1$ depending on the ordering of $\widetilde{{\mathbf {x}}}_{i,1},\widetilde{{\mathbf {x}}}_{i,2}$ as endpoints of $\widetilde{\ell }_i$. Note that, because of the last equality in (6.2b), $\widetilde{{\mathbf {P}}}_{\Gamma }$ for Option II can be reorganized as $\widetilde{{\mathbf {P}}}_{\Gamma } \in {\mathbb {C}}^{\widetilde{N}_\text {nodes}^\text {bnd}, N_{+}}$ (degrees of freedom on the boundary nodes of $\widetilde{\mathcal {M}}$).

Option II, i.e., the discrete system (4.12) with matrices $\widetilde{{\mathbf {P}}}_{\Gamma }$ of (6.2b) and ${\mathbf {M}}_{+}$ of (5.5), is used for the numerical results of Sect. 6.3 for the eddy-current model of Sect. 1.2. Here we specialize the discrete system (4.12) for that eddy-current model, using CM and a Trefftz method for discretization:

6.3 $$\begin{aligned} \begin{pmatrix} ({\mathbf {D}}^1)^{\mathsf {T}}{\mathbf {M}}_{\nu }{\mathbf {D}}^1 - \imath \omega {\mathbf {M}}_{\sigma } &{} ({\mathbf {T}}_{\Gamma }^1)^{\mathsf {T}}\widetilde{{\mathbf {D}}}_\Gamma ^0\widetilde{{\mathbf {P}}}_{\Gamma } \\ \widetilde{{\mathbf {P}}}_{\Gamma }^{\,{\mathsf {H}}}(\widetilde{{\mathbf {D}}}_\Gamma ^0)^{\mathsf {T}}{\mathbf {T}}_{\Gamma }^1 &{} {\mathbf {M}}_{+} \end{pmatrix} \begin{pmatrix} \vec {{\mathbf {a}}}_\Omega \\ \vec {{\mathbf {v}}}_{+} \end{pmatrix} = \begin{pmatrix} \widetilde{{\mathbf {J}}}_{0} \\ \mathbf {0} \end{pmatrix}, \end{aligned}$$

which has a similar structure to the system described in [36] between CM and the *boundary element method*, being both complex Hermitian.

(6.3) contains the following terms:

- ${\mathbf {D}}^1 \in \left\{ -1,0,1\right\} ^{N_\text {faces}, N_\text {edges}}$ is the incidence matrix from edges to faces of $\mathcal {M}$ in $\Omega$ (*discrete curl*).
- Mass matrices ${\mathbf {M}}_{\nu } \in {\mathbb {R}}^{N_\text {faces}, N_\text {faces}}$ and ${\mathbf {M}}_{\sigma } \in {\mathbb {R}}^{N_\text {edges}, N_\text {edges}}$ for CM were introduced in (6.1).
- ${\mathbf {T}}_{\Gamma }^1 \in \left\{ 0,1\right\} ^{N_\text {edges}^\text {bnd}, N_\text {edges}}$ identifies the boundary edges out of all the edges of $\mathcal {M}$.
- Note that the pairing matrix $\widetilde{{\mathbf {K}}}_{1,\Gamma }^{1}$ of (4.12) does not appear in (6.3), being an identity matrix under CM.
- $\widetilde{{\mathbf {D}}}_{\Gamma }^0 \in \left\{ -1,0,1\right\} ^{\widetilde{N}_\text {edges}^\text {bnd}, \widetilde{N}_\text {nodes}^\text {bnd}}$ is the incidence matrix from boundary nodes of $\widetilde{\mathcal {M}}$ to boundary edges of $\widetilde{\mathcal {M}}$ (*discrete boundary gradient*).
- $\widetilde{{\mathbf {P}}}_{\Gamma } \in {\mathbb {C}}^{\widetilde{N}_\text {nodes}^\text {bnd}, N_{+}}$ has the entries shown in (6.2b).
- $\vec {{\mathbf {a}}}_\Omega \in {\mathbb {C}}^{N_\text {edges}}$ is the vector of line integrals of ${\mathbf {A}}$ on the edges of $\mathcal {M}$.
- $\widetilde{{\mathbf {J}}}_{\Omega ,0} \in {\mathbb {C}}^{N_\text {edges}}$ is the vector of fluxes of ${\mathbf {J}}_0$ through the faces of $\widetilde{\mathcal {M}}$ (with a bijective correspondence to line integrals on the edges of $\mathcal {M}$). Entries of $\widetilde{{\mathbf {J}}}_0$ are non-trivial only when related to dual faces in the source domain $\Omega _0$ and are computed with the algebraic procedure proposed in [35], which makes it possible to construct a divergence-free source current vector within $\Omega _0$.
- The energy matrix ${\mathbf {M}}_{+}\in {\mathbb {C}}^{N_{+}, N_{+}}$ of (5.5) and the vector of Trefftz coefficients $\vec {{\mathbf {v}}}_{+} \in {\mathbb {C}}^{N_{+}}$ need no further explanations.

### Results

The CM–Trefftz coupling is implemented in a vectorized MATLAB code and tested with a realistic 3D eddy-current problem, which consists of a cylindrical inductor (made of a conductive material) excited by an alternating current-driven coil. To assess the accuracy of the 3D CM–Trefftz method, we also solved this axisymmetric model (on a radial cross-section) with a third-order 2D FEM approximation, computed with COMSOL Multiphysics 5.5, and used that solution as reference. All our numerical tests were run on a laptop with an Intel Core i7-6920HQ processor (2.90 GHz clock frequency) and 16 GB of RAM.

**Fig. 1:**
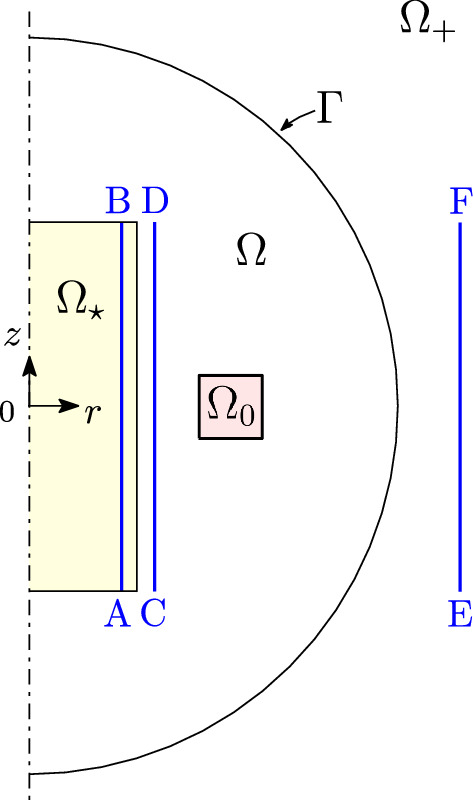
Axisymmetric inductor model: $\Omega _\star$ is the core domain (in yellow), $\Omega _0$ the coil domain (in pink). $\Omega$ is the domain discretized by CM and $\Omega _{+}$ the exterior Trefftz domain. To assess the local accuracy of our method, the eddy-current density is computed along the line A–B ($r=9\,\hbox {mm}$, $z=\left[ -20,20\right] \,\hbox {mm}$), while the magnetic flux density is computed along the line C–D ($r=11\,\hbox {mm}$, $z=\left[ -20,20\right] \,\hbox {mm}$) and along the line E–F ($r=45\,\hbox {mm}$, $z=\left[ -20,20\right] \,\hbox {mm}$) (color figure online)

Figure 1 shows a sketch of the axisymmetric inductor (radial cross-section). The cylindrical inductor core $\Omega _\star$ (10 mm radius, 40 mm high) is made of a conductive material with $25\,\hbox {MS m}^{-1}$ conductivity and 2 relative permeability. The inductor coil $\Omega _0$ (square cross-section, 4 mm side, centered at $r=15\,\hbox {mm}$, $z=0$) carries a 16 A current at 200 Hz frequency, which excites eddy currents in $\Omega _\star$. Finally, the surrounding domain $\Omega$ is a sphere with 40 mm radius, which includes an air domain (null conductivity, 1 relative permeability). $\Omega$ is discretized by CM (with piecewise-constant edge and face elements) using a tetrahedral mesh.

With a Trefftz method, meshing the exterior domain $\Omega _{+}$ is not required, since the magnetic field there is approximated by the gradients of (5.4). This Trefftz discretization is coupled with CM by suitable transmission conditions at the interface $\Gamma$.

The eddy-current distribution in $\Omega _\star$, the magnetic flux density distribution in $\Omega$, i.e., the real and imaginary parts of the *r*- and *z*-field components, and the aforementioned accurate numerical solution are computed with third-order 2D FEM as reference. To account for the truncation error, the FEM model geometry is bounded by a large circle (600 mm radius) on which radiation boundary conditions are applied. The whole 2D FEM domain is partitioned into 30 797 third-order triangles.

For the 3D CM–Trefftz coupling, tetrahedral meshes with different *mesh sizes* *h* (reported in table 1) are employed to check the convergence of the CM–Trefftz method. We define mesh size as the maximum diameter of spheres circumscribing the tetrahedrons of a mesh. The same fixed Trefftz space is used with different mesh refinements: one expansion (5.4) of fifth order and centered at the origin is used. In fact, it is observed that increasing the order does not provide an improvement in the convergence of the error (i.e., the CM error dominates) and the local magnetic field reconstruction is very good even with a low order.

For the sake of comparison, the following relative errors (in $L^2$-norm) for domains $\Omega _\star$ and $\Omega$ are defined:

6.4 $$\begin{aligned} e_{{\mathbf {J}}} = \frac{\Vert {\mathbf {J}}_h-{\mathbf {J}} \Vert _{L^2(\Omega _\star )}}{\Vert {\mathbf {J}} \Vert _{L^2(\Omega _\star )}}, \quad e_{{\mathbf {B}}} = \frac{\Vert {\mathbf {B}}_h-{\mathbf {B}} \Vert _{L^2(\Omega )}}{\Vert {\mathbf {B}} \Vert _{L^2(\Omega )}}, \end{aligned}$$

where ${\mathbf {J}}_h,{\mathbf {B}}_h$ are the approximated field distributions computed by the 3D CM–Trefftz coupling and ${\mathbf {B}},{\mathbf {J}}$ the reference field distributions computed by third-order 2D FEM.

Table 1 shows the assembling and solving times needed for each mesh. The assembling time encompasses the generation of both incidence and mass matrices, the generation of the right-hand side vector (from source currents), and the assembly of the final matrix system (6.3). The solving time corresponds to the solution of the final matrix system, which is carried out by the BiCGSTAB iterative solver [9, p. 24, Section 2.3.8] with a SSOR preconditioner [9, p. 37, Section 3.3] (given a prescribed tolerance of $10^{-10}$).

**Table 1:** Computational times for assembling and solving the final matrix system (6.3)

*h* (mm)	# tests	# DoFs	CPU time (s), assembling	CPU time (s), solving
4.17	229,929	273,818	24.35	35.81
3.18	570,141	675,111	57.01	129.89
2.76	1,122,510	1,325,214	109.98	320.99
2.34	1,512,855	1,783,924	153.33	534.57
2.05	2,679,105	3,153,302	275.93	1092.55

**Fig. 2:**
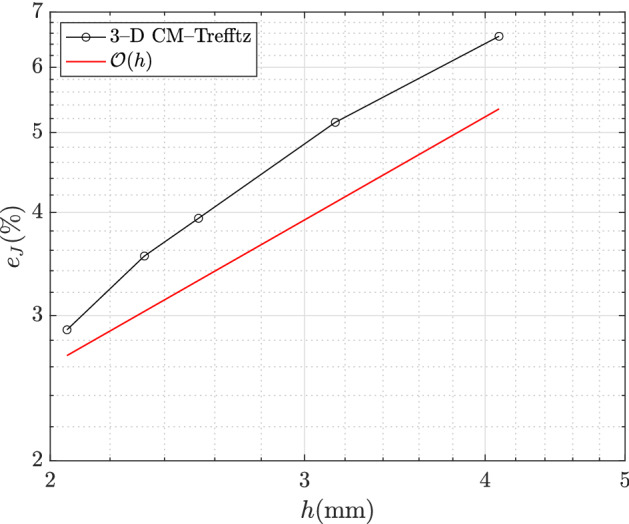
Discrepancy ($L^2$-norm) in $\Omega _\star$ between the eddy-current density of third-order 2D FEM and 3D CM–Trefftz. The dashed line marks first-order convergence $\mathcal {O}(h)$

**Fig. 3:**
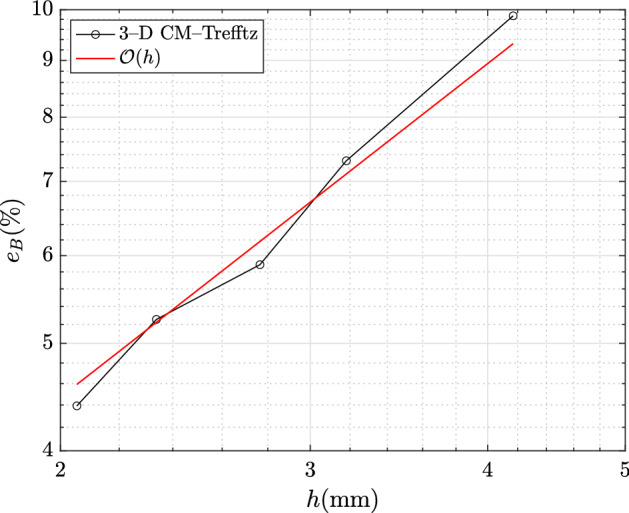
Discrepancy ($L^2$-norm) in $\Omega$ between the magnetic flux density of third-order 2D FEM and 3D CM–Trefftz. The dashed line marks first-order convergence $\mathcal {O}(h)$

Figures 2 and 3 show the relative errors (6.4), computed for the same mesh refinements as above. It can be observed that the 3D CM–Trefftz coupling attains a first-order convergence rate in the $L^2$-norm. In particular, Fig. 2 proves that a very good accuracy in the eddy-current density reconstruction within the core is obtained by using the proposed hybrid approach.

In order to validate the accuracy of CM–Trefftz on a local level, the eddy-current density is computed in the core region along the line A–B in Fig. 1, with coordinates $r=9\,\hbox {mm}$ and $z=\left[ -20,20\right] \,\hbox {mm}$. In order to assess the accuracy of both methods separately, the magnetic flux density in the air region is computed both in the CM (along line C–D in Fig. 1, $r=11\,\hbox {mm}$, $z=\left[ -20,20\right] \,\hbox {mm}$) and Trefftz domains (along the line E–F in Fig. 1, $r=45\,\hbox {mm}$, $z=\left[ -20,20\right] \,\hbox {mm}$).

In the following, field lines with 3D CM–Trefftz are considered on the symmetry plane $y=0$ (but other radial cross sections can be considered). Moreover, comparisons between field profiles are all taken with the intermediate mesh refinement of $h=2.76\,\hbox {mm}$.

**Fig. 4:**
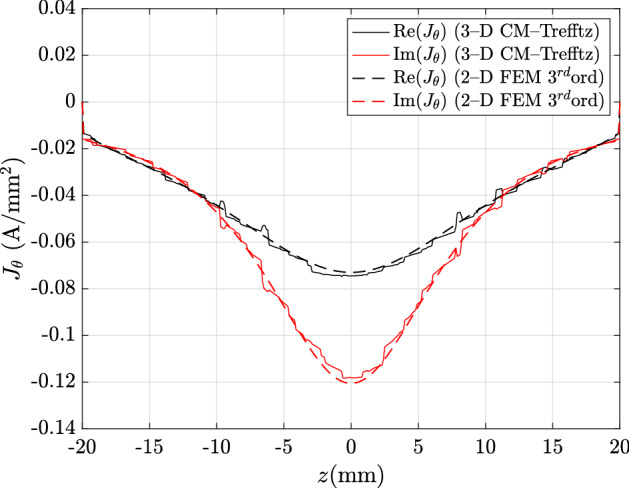
Real and imaginary parts of ${\mathbf {J}}_\theta$ along line A–B in Fig. 1: CM–Trefftz results for mesh size $h=2.76\,\hbox {mm}$. The plot of 3D CM–Trefftz is marked by the straight line, third-order 2D FEM by the dashed line

Figure 4 shows the real and imaginary parts of the azimuthal eddy-current density ${\mathbf {J}}_\theta$ along line A–B. The CM–Trefftz plot is obtained by locally interpolating the electric field (proportional to the eddy-current density) with piecewise-constant edge basis functions. Maximum discrepancies from the third-order 2D FEM solution for the real and imaginary parts of ${\mathbf {J}}_\theta$ are 18.36% and 13.14%, respectively.

**Fig. 5:**
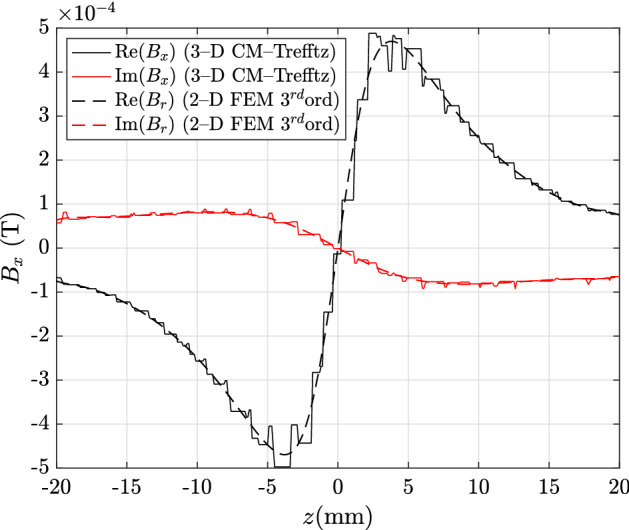
Real and imaginary parts of ${\mathbf {B}}_x$ along line C–D in Fig. 1: CM–Trefftz results for mesh size $h=2.76\,\hbox {mm}$. The plot of 3D CM–Trefftz is marked by the straight line, third-order 2D FEM by the dashed line

**Fig. 6:**
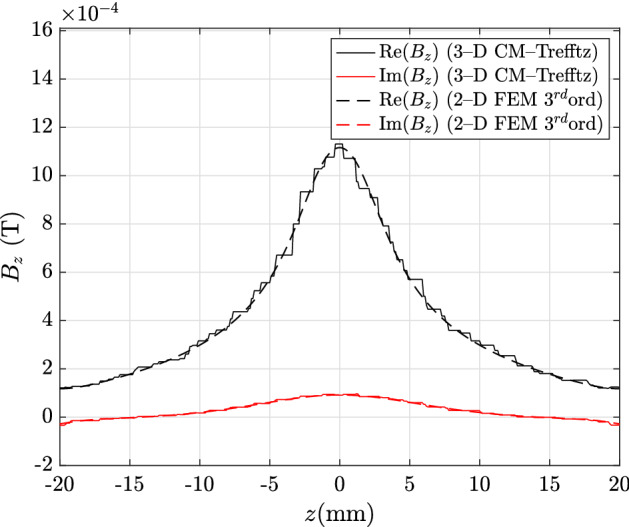
Real and imaginary parts of ${\mathbf {B}}_z$ along line C–D in Fig. 1: CM–Trefftz results for mesh size $h=2.76\,\hbox {mm}$. The plot of 3D CM–Trefftz is marked by the straight line, third-order 2D FEM by the dashed line

The same comparison is then extended to the magnetic flux density in the domain with CM discretization. In this case, CM–Trefftz plots are obtained by locally interpolating the magnetic flux density with piecewise-constant face basis functions. Figures 5 and 6 show the real and imaginary parts of the *x*- and *z*-components along line C–D. Maximum discrepancies are 26.06%, 20% for the real and imaginary part of ${\mathbf {B}}_x$, and 8.48%, 11.77% for the real and imaginary part of ${\mathbf {B}}_z$.

**Fig. 7:**
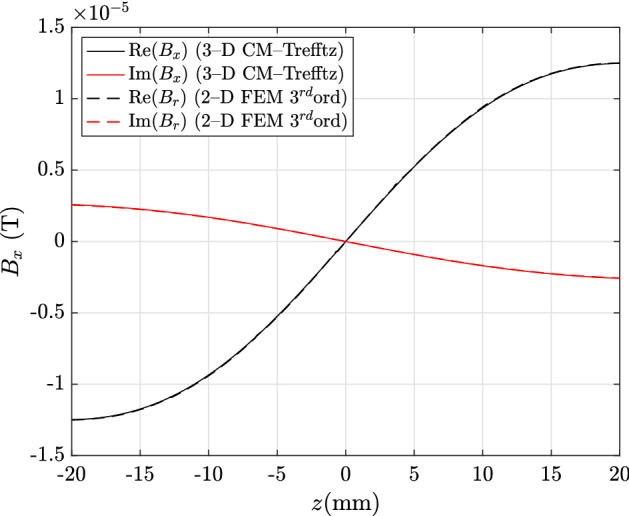
Real and imaginary parts of ${\mathbf {B}}_x$ along line E–F in Fig. 1: CM–Trefftz results for mesh size $h=2.76\,\hbox {mm}$. The plot of 3D CM–Trefftz is marked by the straight line, third-order 2D FEM by the dashed line

**Fig. 8:**
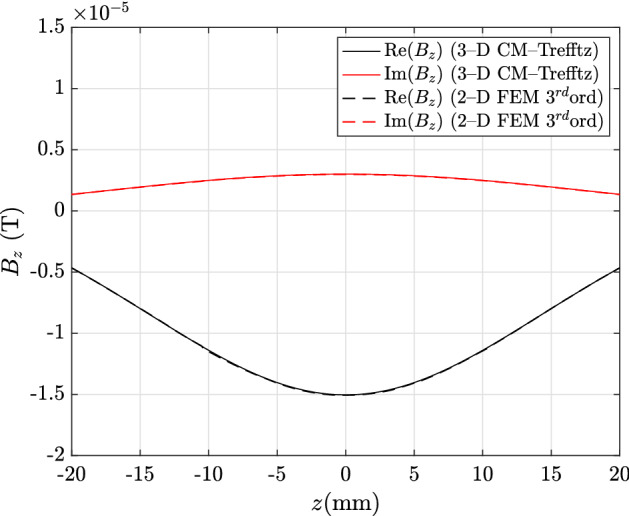
Real and imaginary parts of ${\mathbf {B}}_z$ along line E–F in Fig. 1: CM–Trefftz results for mesh size $h=2.76\,\hbox {mm}$. The plot of 3D CM–Trefftz is marked by the straight line, third-order 2D FEM by the dashed line

In the exterior domain, with Trefftz discretization, the hybrid approach proves to be highly accurate, even though a single expansion to the fifth order is used. For plots shown in Figs. 7 and 8, with field profiles computed along line E–F, all discrepancies are under 1%: 0.81%, 0.86% for the real and imaginary part of ${\mathbf {B}}_x$, and 0.73%, 0.95% for the real and imaginary part of ${\mathbf {B}}_z$.

## Conclusions

The authors are not aware of any prior work addressing the coupling of Trefftz methods with a general framework for numerical schemes based on volume meshes, like co-chain calculus. We explain for the first time how to formulate an interface operator for this framework that takes into account the exterior problem (see (4.8)). The particular case of coupling the cell method with Trefftz basis functions is also presented here for the first time.

The other particular case of Trefftz methods coupled with the finite element method as mesh-based discretization can be seen in [18], which also includes numerical results. There one can see that, compared to other hybrid approaches that rely on the boundary element method, using a Trefftz method enjoys advantages [29, p. 51], namely

- a simpler assembly process, as there are no singular integrals, and
- an exponentially-convergent approximation error when the unknown possesses an analytic extension beyond the Trefftz approximation domain (see Sect. 3).

Admittedly, Trefftz methods can suffer from ill-conditioning. However, the impact of ill-conditioning is mitigated due to the low number of degrees of freedom required for Trefftz methods, given their exponential convergence. The dense Trefftz blocks in the coupling matrices are therefore small and those degrees of freedom may even be eliminated by a Schur complement approach (see (4.7)).

## Abbreviations

FEEC: Finite element exterior calculus
DEC: Discrete exterior calculus
DtN: Dirichlet-to-Neumann
CM: Cell method
DoF: Degree of freedom

## Footnotes

1 This also translates into a condition on the *l*-th Betti number of $\Omega$.

2 This also translates into a condition on the 2nd Betti number of $\Omega$.

3 We refer to [23, p. 271, Definition 1] for the conditions that a sequence of spaces of differential forms has to satisfy to be discretizable.

4 Given a numerical method expressed by co-chain calculus, some of its degrees of freedom may be represented on the primary mesh and others on the secondary mesh. In this case, a bijective relationship between the two types of unknowns is needed, which can be achieved by using a secondary mesh *dual* to the primary mesh. This leads to numerical schemes fitting the framework of DEC.

5 Note that the identification of discrete spaces with mesh facets is restricted to lowest-order methods (for example, lowest-order Whitney forms). In fact, co-chain calculus does not generalize easily to higher-order spaces.

6 ${\mathbf {M}}_\alpha ^l,{\mathbf {M}}_\gamma ^{l-1}$ can also be invertible matrices scaled by a negative or imaginary scalar. Imaginary $\gamma$ is indeed the case of (1.2b).

7 While the computation of the entries of matrices ${\mathbf {M}}_\alpha ^l,{\mathbf {M}}_\gamma ^{l-1}$ is done differently in FEEC and DEC, as discussed in [23, p. 277, Section 5], the discrete matrix forms of co-chain calculus only need to respect the few algebraic requirements listed here, independent of the details of the approximation. Hence, the actual construction of such matrices is only addressed when the framework of co-chain calculus is specialized into a numerical method (e.g., Sect. 6.1).

8 Note that pairing matrices, like mass matrices, depend on the adopted numerical method (i.e., on the choice of the discrete basis functions that approximate the differential forms).

9 If the boundary value problem is nonhomogeneous, then the right-hand side can be taken into account by a Green’s function that acts as known term of the Trefftz discretization.

10 This is the choice followed in Sect. 5, where we give examples of Trefftz basis functions for the eddy-current problem of Sect. 1.2.

11 Hence, Trefftz functions are smooth and must be available as closed-form analytic expressions together with all their derivatives.

12 Furthermore, an artificial boundary can always lead to the simply-connected $\Omega _{+}$ required for the simplified exterior problem (1.10).

13 If $\gamma =0$, we further impose the gauge condition $\mathrm {d}\left( \star \,v\right) =0$ in (3.1a).

14 For $m=1$ as in Sect. 1.2, therefore $\mathcal {T}^{0}(\Omega _{+}) \in \Lambda ^{0}(\Omega _{+})$, the gauge condition is always satisfied because $\star \,v$ would be a 3-form, whose exterior derivative always vanishes.

15 The variational problem (4.11) is related to the hybrid coupling proposed in [2, 3].

16 The construction of $\widetilde{{\mathbf {P}}}_{\Gamma }$ also involves the mesh-based discretization of co-chains (2.1): an example will be given in Sect. 6.2 for the cell method as mesh-based discretization.

17 Note that functions (5.1) fulfill the Coulomb gauge.

18 Note that CM establishes the relationship $N_{l-1}=\widetilde{N}_{m}$, which leads to the pairing matrix $\widetilde{{\mathbf {K}}}_{m,\Gamma }^{l-1}$ becoming an identity.

19 In fact, Stokes’ theorem [28, p. 31] allows to rewrite differential operators applied to fields in integral form with incidence matrices between integrals on entities of the (primary and dual) meshes.
